# Reinforcement Learning for Traversing Chemical Structure Space: Optimizing Transition States and Minimum Energy Paths of Molecules

**DOI:** 10.1021/acs.jpclett.3c02771

**Published:** 2024-01-03

**Authors:** Rhyan Barrett, Julia Westermayr

**Affiliations:** † Institute of Chemistry, Faculty of Chemistry and Mineralogy, University of Leipzig, Johannisallee 29, 04103 Leipzig, Germany; ‡ Center for Scalable Data Analytics and Artificial Intelligence (ScaDS.AI), Dresden/Leipzig, Humboldtstraße 25, 04105 Leipzig, Germany

## Abstract

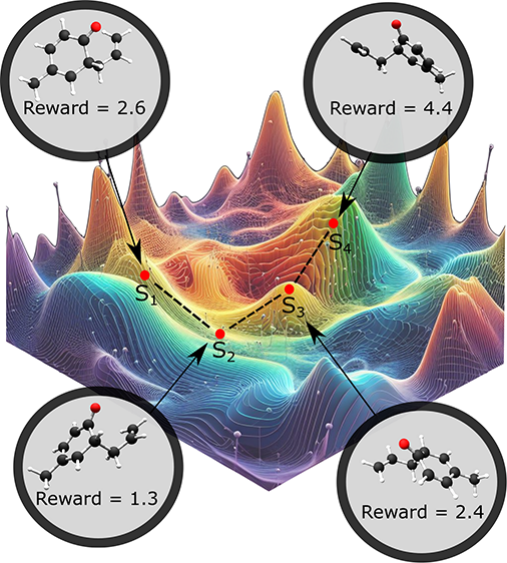

In recent years, deep learning has made remarkable strides, surpassing human capabilities in tasks, such as strategy games, and it has found applications in complex domains, including protein folding. In the realm of quantum chemistry, machine learning methods have primarily served as predictive tools or design aids using generative models, while reinforcement learning remains in its early stages of exploration. This work introduces an actor–critic reinforcement learning framework suitable for diverse optimization tasks, such as searching for molecular structures with specific properties within conformational spaces. As an example, we show an implementation of this scheme for calculating minimum energy pathways of a Claisen rearrangement reaction and a number of S_N_2 reactions. The results show that the algorithm is able to accurately predict minimum energy pathways and, thus, transition states, providing the first steps in using actor–critic methods to study chemical reactions.

In recent years, machine learning (ML) has had a large impact on society in many different areas, from large language models,^1−3^ such as ChatGPT,^4^ to various applications in the financial sector.^5−7^ However, ML has only recently found its way to subject areas outside of a computational or financial setting. In physics and chemistry, some areas of ML can still be considered in their infancy. Initial works in ML for chemistry mainly look at predicting primary outputs, such as wave functions^8,9^ or electron density,^10,11^ secondary outputs, such as energies, forces, or dipole moments,^12−18^ and tertiary outputs, such as reaction rates^19,20^ or fundamental gaps^21−23^ of quantum systems, using supervised ML. These developments have allowed scientists to calculate electronic and other properties at a much larger speed and, hence, lower computational cost than the associated quantum mechanical reference methods. In a general setting, the task of predicting properties of a quantum system or, in principle, any predictive task involves constructing a function that maps a parameter space to a low dimensional property space, such as energy or forces, or to a higher dimensional property space, for example, the parameters of a wave function or excited states.^24−26^ This function aims to closely approximate the true properties of the system. Current quantum chemical methods operate on the basis of a similar principle. In these methods, the objective is to build a parametrized wave function or probability density that minimizes the energy of the quantum system using the variational principle.^27^ For each new molecular structure, another optimization of the wave function must be performed. A general mapping that adequately describes the relationship between all molecular structures and their associated properties remains unknown.

Neural networks are known to be able to model any relationship^28^ as a result of their large amount of learnable parameters, which allows them to construct mappings between almost any two quantities. Thus, in theory, they should be able to construct this mapping given the relevant data set. Current work in quantum chemistry looks at encoding useful physical properties into neural network architectures^15,29−32^ to allow ML models to more easily predict the molecular properties from the structures using less learnable parameters.

The problem of finding these mappings can also be formulated slightly differently; rather than optimizing parameters with a currently existing optimization algorithm, we could look to have another neural network to predict the best fitting parameters. To do this, one can think about the process of changing parameters as a game, where, at each step in the game, the parameters are adjusted. A neural network can then explore the game to find the best neural network parameters for our problem. The task of adapting parameters in a game is the idea behind reinforcement learning. The advantage of using reinforcement learning in comparison to a normal optimization process is that a larger amount of exploration is obtained in the parameter space. In particular, reinforcement learning has already been shown to be effective in molecular design and generation,^33−36^ crystal and surface structure determination,^37,38^ identifying retrosynthetic pathways of molecules,^39^ and advancing experiments,^40^ especially in the context of automation of synthesis.^41,42^

An additional area where the optimization of parameters is of interest is the space of different molecular structures, where one wishes to optimize the positions of atoms against some reward function. Some examples are geometry optimization, minimum energy pathway calculation, or the search for critical points on excited-state potential energy surfaces, such as conical intersections. In this work, we explore the application of reinforcement learning to this task and test it in the search for minimum energy pathways and transition states.

A scheme of the algorithm developed in this work is illustrated in Figure 1. The method behind the reinforcement learning algorithm can be expressed as a Markov chain and will be explained in the context for molecules in the following text. Initially, a molecular conformation is taken, and the atomic positions are adapted with an action constructed by the neural network. The reward *r*_*i*_ is then calculated and fed back to the neural network so that it can re-evaluate its decision to improve in the future. More precisely, the definitions of the different components that are to be defined for this task and in the context of molecular systems are summarized and described as follows:

**Figure 1:**
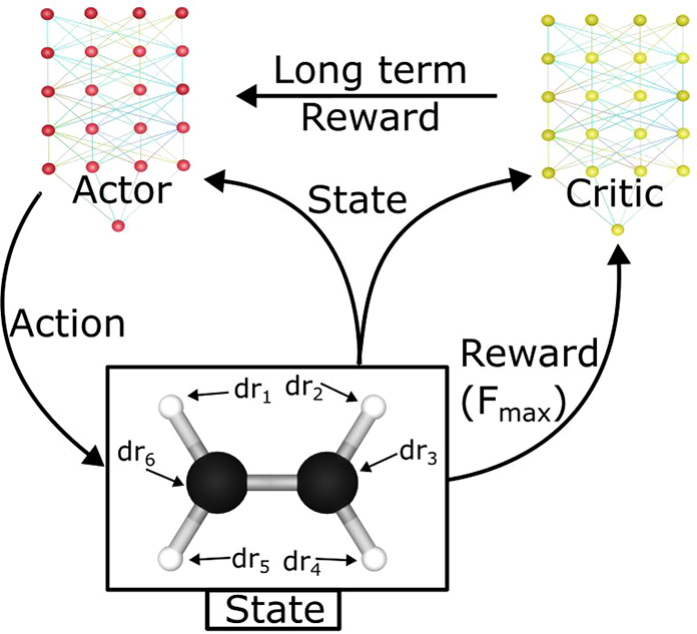
Actor–critic workflow. The actor constructs an action which alters the positions of the atoms in the molecule. The current configuration of the molecules is known as the state. A reward is calculated using the new configuration which is passed to the critic so that the long-term reward can be estimated. The long-term reward and current state are then used in the optimization of the actor’s policy. Given the updated policy the process is then repeated.

State: The current conformation of a molecule can be described by atomic numbers, *Z*_*i*_, and atomic positions, *R*_*i*_ ∈ **R**^3^.
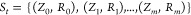
1

Action: Given the atomic numbers and atomic positions of the current molecule, the set of corresponding new positions of the atoms in the molecule can be constructed. The new atomic positions, *R*_*i*_ + δ*R*_*i*_, are then constructed as follows:
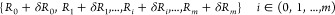
2The δ*R*_*i*_ values are generated by the neural network.

Reward: The reward is used to let the reinforcement learning agent assess the success of its actions and is dependent upon the particular task at hand. In the case of geometry optimizations, the task would be, for instance, to maximize *E*_*t*_^–1^ to obtain the minimum energy structure.

The reward calculated is defined as immediate feedback after a change in the current state. However, in this case, the quantity of interest is the long-term reward or the reward given by the final structure. Because the long-term reward is not known until the task is complete, an estimation of an expected long-term reward given by the current state is calculated. Two main methodologies in reinforcement learning have been developed for this purpose,^43^ namely, value-based methods, such as *Q* learning,^44^ and policy-based methods, such as the actor in actor–critic methods.^45^ We will use actor–critic methods in this paper which combines the policy-based actor and value-based critic.

The actor–critic method^46,47^ used in this work is a class of reinforcement learning shown to be effective in high-dimensional problems,^48,49^ with which will be dealt with in many situations in chemistry, particularly in the case of large molecules. This method involves three components: a state, an actor that interacts with the environment, and a critic, which evaluates how well the actor performed. This concept is illustrated in Figure 1. The objective of the actor is to learn a policy to maximize long-term rewards, and the critic attempts to predict the long-term reward, *V*(*S*_*k*_), given the previous behavior of the actor.

More precisely, looking at each step, the actor receives a molecule, the state, that is, the result of the action performed on the environment. In this work, the action consists of changes in the current atomic positions of a molecule. The reward of the given state is calculated using some evaluation metric, i.e., the energy of the new state or the corresponding forces. This reward along with the state is passed to the critic, which then estimates what the expected long-term reward produced by the actor will be given the current state. The expected long-term reward is the core quantity in actor–critic models from which the actor learns to adjust itself. However, this quantity is not straightforward to calculate, because it would be needed to calculate all possible adaptations from a particular molecular conformation to then take the final reward. To avoid this tedious task, a neural network is used to estimate this quantity.

The rewards calculated from the adjustments of a molecule form a random walk, as illustrated in Figure 2. The random walks show how an actor might change the atomic position along with the associated rewards from the initial molecular conformer *S*_*k*_. The straight line represents the estimate of the critic of the long term; if the starting state is *S*_*k*_, then this value is written as *V*(*S*_*k*_). However, this scheme can prove quite inefficient because every critic update must be performed at the end of the episode, once the long-term reward has been calculated. To provide an approximation of *V*(*S*_*k*_) before the end of the episode, a process called temporal difference learning is used. Temporal difference learning is illustrated in Figure 2. It involves breaking down an episode into smaller pieces and then looking at the final reward received plus the estimate from the critic at the final state in the section. In this way, the actor and critic can update themselves more frequently.

**Figure 2:**
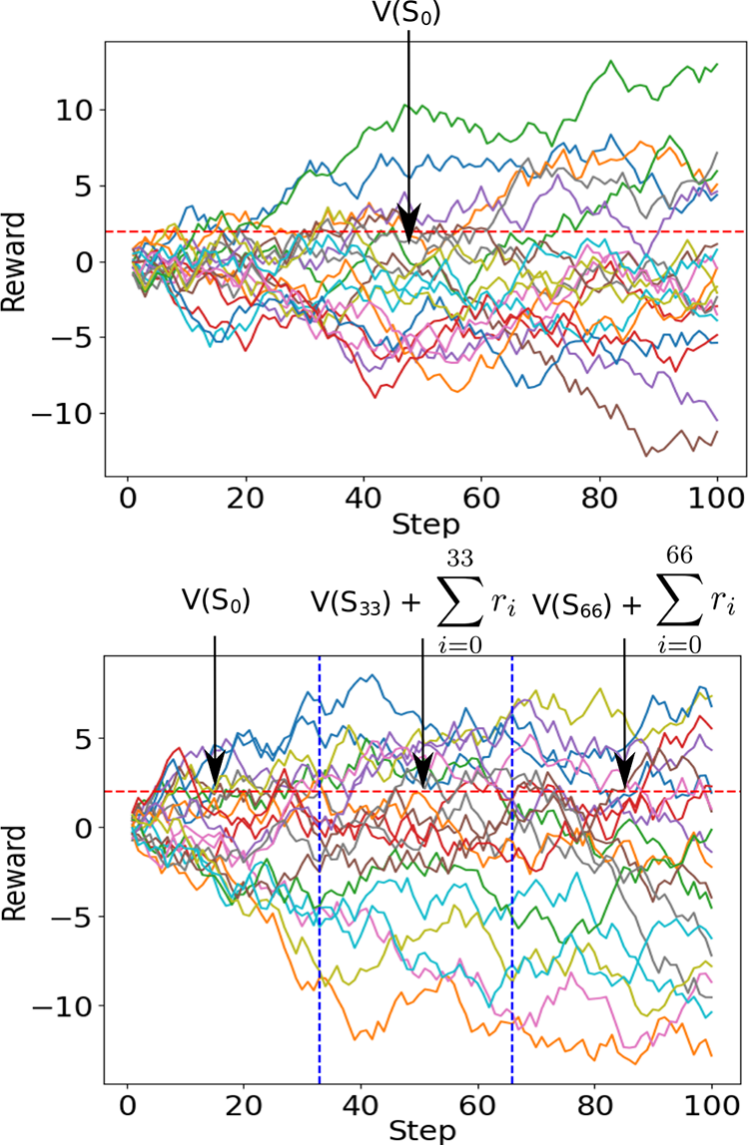
Possible rewards generated by an actor–critic algorithm. (a) The solid lines represent how the reward evolves over the episode, and the dotted line represents the estimate of the critic at step *S*_0_, *V*(*S*_0_). (b) Again, the solid lines represent how the reward evolves over the whole episode. The episode can then be broken up into several pieces; in each segment, an estimation of the expectation is calculated. In the first segment, the expectation is the estimate of the critic at state *S*_0_;at step *S*_33_, it is given by the critic estimate at step 33 plus the previous rewards; and finally, in the final segment, the estimate is the estimate of the critic at step 66 plus the previous rewards.

To define mathematically, the cumulative rewards received through the episode, *R*_*k*_, can be rewritten
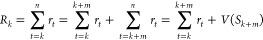
3with *r*_*t*_ being the rewards of each individual step. As seen above, the total rewards received are split through the episode into two sums: one for the smaller initial episode and then the sum for the remainder of the episode, which is approximated by the critic. This value is used to update the critic.

Now that an estimate of the long-term rewards of a particular molecular state has been discussed, it can be integrated with the agent so that better decisions are obtained. As mentioned earlier, there are two main approaches, but the focus will be on actor–critic methods, a policy- and value-based method. Actor–critic methods operate by taking the estimated long-term reward using the critic and measuring if the decisions of the actor lead to a value higher or lower than the expected long-term reward. The advantage can be written as
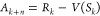
4If *A*_*k*+*n*_ > 0, then the decisions taken by the actor can be considered as positive, because they lead to higher rewards. This, in turn, leads to these actions being positively reinforced in the actor. On the contrary, if *A*_*k*+*n*_ < 0, this corresponds to the actor-selecting actions, which lead to lower rewards. Over time, the actor should learn the steps that lead the maximization of the reward function by selecting actions with positive advantages, i.e., higher final rewards.

Now that the overall structure of the actor–critic algorithm has been described, we can consider how to implement this in the case of molecular structures. The decisions of the actor can be either deterministic or stochastic in nature; i.e., either the actor output is the new positions of the atoms or a probability distribution from which a new position is sampled. The second case is preferable because it enforces more exploration and, thus, a higher likelihood of finding an optimal solution. This is the method that is implemented in this paper. Additionally, we can divide the expected long-term reward estimated by the critic into atomic contributions allowing us to use this for arbitrary-sized molecules. The mathematical details of both the actor and critic will be left to the Supporting Information, including an additional description in section S1. Because an overview of the actor and critic system has been described, we will attempt to apply this to the case of minimum energy pathway prediction as an application of actor–critic methods in a molecular setting.

Conventional methods to compute minimum energy pathways require many sequential evaluations of the quantum Hamiltonian, leading to high computational costs and as a result of the vastness and intricate local topological structure of the potential energy surface, usually requiring a lot of human input to successfully converge. Therefore, ML is promising to advance this field, but only few works exist in this direction.^50−53^ One work is TS-Net,^50^ which applies a tensor-field network to predict the structure of a transition state. However, this method requires a training set with transition states, and the generation of transition states for a training set is computationally expensive and time-consuming, thus limiting the applicability of this model, especially when targeting large systems. Another work reformulates the transition state search into a shooting game using reinforcement learning techniques.^51^ This technique is related to a common computational workflow, namely, transition path sampling, and operates by choosing a coordinate in phase space from which two trajectories are started with opposite momenta. If the trajectories reach the desired products and reactants, the episode is considered successful. While this approach is powerful in theory, it requires Monte Carlo techniques^54^ to identify promising pathways before training. As a consequence, this method can become highly expensive as the molecular systems become larger. Thus, to study larger systems, a faster way of performing quantum mechanical calculations is required along with an intelligent search of the potential energy surface.

To improve on the methods mentioned, the developed model is based on the nudged elastic band method (NEB)^55,56^ commonly used in quantum chemistry to predict minimum energy pathways. NEB involves dividing the reaction pathway into a series of discrete images or ”beads”. Each bead represents a possible intermediate state in the reaction path. Given a reaction pathway, the total force on each image is determined by a combination of the internal interatomic forces of each image perpendicular to the reaction pathway, *F*_int_⊥, and the virtual harmonic spring forces holding the images together parallel to the reaction pathway, *F*_spr_∥. This total force is quantity to be minimized to approximate the minimum energy pathway. More precisely, the set of forces on the atoms in image *j* can be alternatively described mathematically
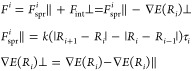
5where *R*_*i*_ represents the positions of atom *i* in image *j*, τ_*i*_ is the tangent to the reaction path at image *j*, and *k* is the spring constant controlling the strength of the harmonic springs. Using this, the maximum force *F*_max_ on any one atom in any image along the reaction pathway can be computed. Here, the aim is to optimize the molecular pathways by adapting molecular configurations in a way that minimizes *F*_max_.

With the NEB algorithm and the actor–critic model derived above kept in mind, the task of finding the minimum energy pathway can be formulated as a reinforcement learning task. In a way similar to the NEB method, at each step in the process, the atomic positions of the molecules on the reaction pathway are modified. This differs from the previous descriptions because a set of molecular structures needs to be considered in contrast to a single molecular structure. The full derivation of this will be left in the Supporting Information; see section S5. As a result of the extremely large configuration space made up by the images along the reaction pathway, a subset of configurations is constructed, which provides a good starting point. To do this, the *F*_int_⊥ and *F*_spr_∥ values for the atoms in each image are constructed using a pre-trained ML model, in our case a model containing the PaiNN deep learning representation.^13^ PaiNN is a polarizable atom interaction neural network that learns equivariant representations in addition to the relation of these features to output targets. Then, for each image, a linear combination of these values is generated to form the desired subspace of potential moves. Analogous to the above, the reinforcement learning algorithm can again be broken down into a series of components. The state is represented by molecules along the reaction path described by atomic numbers and atomic positions of the initial, intermediate, and final structures or images. Thus, the state space consists of the atomic positions of the conformations along the reaction pathway, with their associated atomic positions and atomic numbers. The action as mentioned before consists of a linear interpolation of the vectors *F*_int_⊥ and *F*_spr_∥ for an individual image. The new atomic positions can be derived from force values. The objective can be defined as minimizing the force *F*_max_, as shown above. The reward, *r*_*t*_, at each step is then given by (*F*_max_)^−1^. Maximizing the given reward and minimizing the *F*_max_ value are equivalent.

For the actor and critic to achieve accurate predictions, it is important to convert the Cartesian coordinates into a representation that is both rotationally and translationally invariant.^13^ This can be accomplished through the use of the PaiNN deep learning representation as mentioned before, developed by Schütt et al.^13^ On the basis of this representation, the aim is to predict changes to the positions of atoms in each image along the reaction pathway at each step with the goal to move closer to the minimum energy pathway. Around each atom in the molecule, the contribution of the *F*_int_⊥ and *F*_spr_∥ values is predicted to the linear combination of the total force. A distribution for these contributions is then constructed by the model for the total force, and the new atomic position is sampled from the respective distribution. An illustration of this and how the associated model is built can be seen in Figure 3. The full details of this can be found in section S2 of the Supporting Information. Additionally, details of the training procedure and loss can be found in section S1 of the Supporting Information.

**Figure 3:**
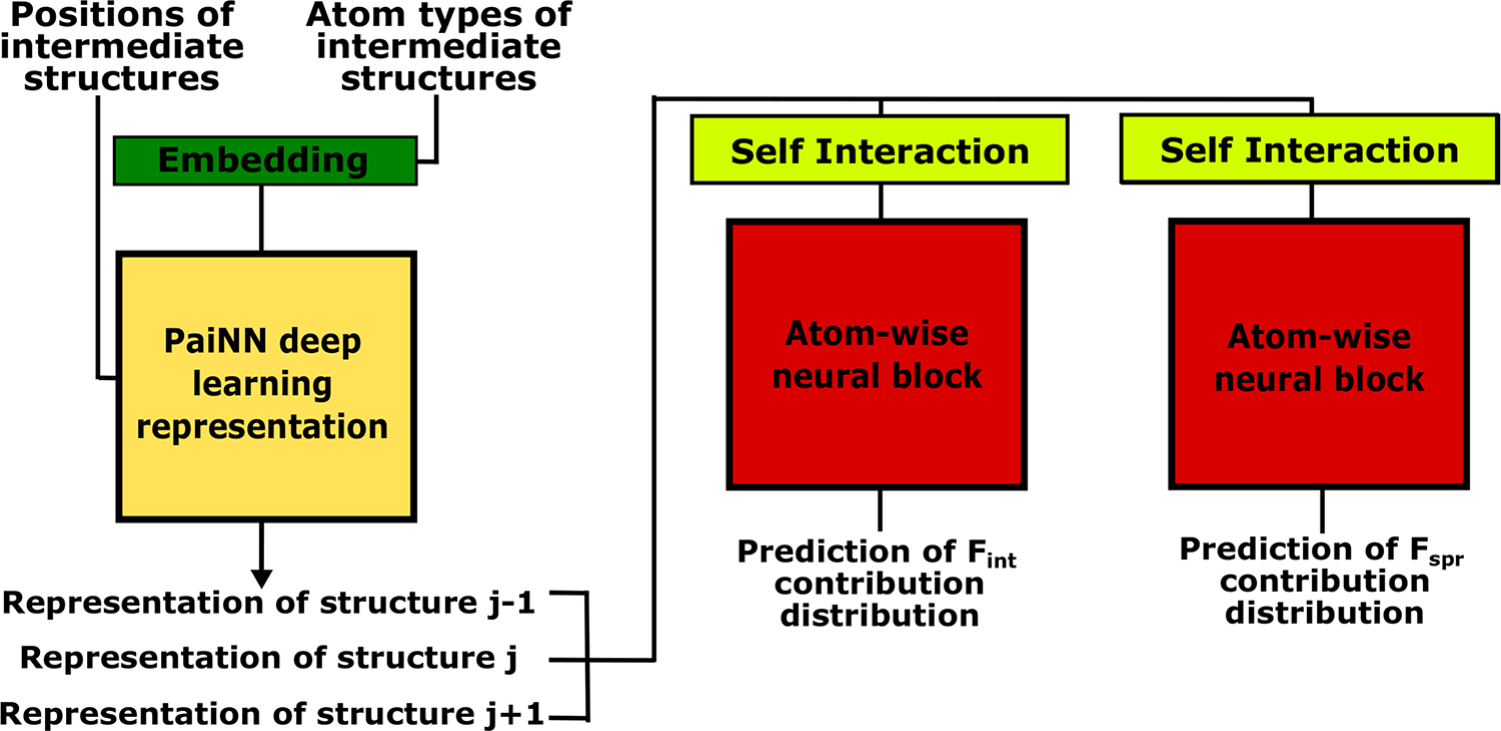
Actor architecture. The PaiNN representation forms the initial feature vectors for the actor. The positions of each atom in the current image are passed through a series of different atom-wise neural layers to produce the respective distributions for the contribution to the total force. The two distributions are then sampled to produce the change in atomic positions at the current time step. A more detailed illustration of the network architecture is given in Figure S4 of the Supporting Information.

The critic model shares the same PaiNN representation layers as the actor model, but instead, the final layers are used to provide an estimate of the value function. In the final layers of the critic, we sum over all of the contributions for each individual atom to obtain the estimate. An overview of the structure is shown in Figure S1 of the Supporting Information, including a more detailed description in section S3.

The performance of the method is tested on a series of test reactions, namely, the allyl-*p*-tolyl ether Claisen rearrangement reaction and multiple reactions obtained from the S_N_2 data set comprising chemical reactions of the form X^–^ + H_3_C–Y → X–CH_3_ + Y^–^, with X, Y ∈ {F, Cl, Br, I} and X ≠ Y.

First, the model is tasked to target the pathway of the allyl-*p*-tolyl ether Claisen rearrangement reaction. As illustrated in Figure 4a, the Claisen rearrangement takes place through a concerted mechanism in which a C–C bond forms between the C1 position of the allyl group and the *ortho* position of the benzene ring (marked as C5) at the same time that the C3–O bond of the ether breaks. This rearrangement initially produces a non-aromatic intermediate, which quickly undergoes a proton shift to reform the aromatic ring in the product. Claisen rearrangement occurs in a six-membered, cyclic transition state involving the concerted movement of six bonding electrons in the first step. The full path of the reaction is attached as Supporting Information.

**Figure 4:**
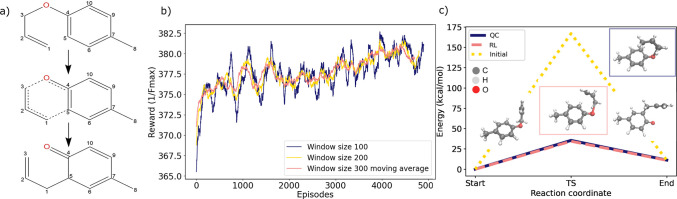
Claisen rearrangement reaction. (a) Illustration of the start, intermediate, and end structures in the allyl-*p*-tolyl ether Claisen rearrangement reaction. (b) Rewards and episode number over the course of the training phase representing the moving average reward values with three respective window sizes. (c) Energies and structures of start and end geometry and found transition state (TS) obtained with quantum chemistry (QC, blue), actor–critic model (red), and initial guess (yellow) from geodesic interpolation.

To assess the reward, a PaiNN^13^ model was trained on a data set containing structures of allyl-*p*-tolyl ether obtained from metadynamics simulations taken from ref (14) to form the initial representation input for the model. The mean absolute errors (MAEs) for energies and forces are 0.22 and 0.37 kcal mol^–1^ Å^–1^, respectively, on a hold-out test set (details on training of PaiNN models are in the Supporting Information in sections S4.1 and S4.2 and Figure S2). Following this, an initial guess is used of the reaction pathway obtained via geodesic interpolation. For consideration of computational efficiency, the model is allowed to sample 10 episodes of length 50 to find a pathway of lower *F*_max_ value as the new initial starting guess. The agent is now trained from this starting guess to minimize the *F*_max_ values. Exact implementation details can be found in the Supporting Information in section S4.4.

The training process can be followed in Figure 4b that illustrates a consistent increase in the reward function and, thus, a decrease in the associated *F*_max_ value. Figure 4c shows the different energy curves and transition states found with the model and quantum chemistry (QC) using standard NEB with density functional theory (DFT) at the PBE0-D4/def2-TZVP level of theory. The activation energy obtained using the model was calculated to be about 37 kcal/mol, which is in very good agreement to the reference value of 35 kcal/mol, especially when considering the initial guess of over 150 kcal/mol.

In the second task, the aim is to minimize the *F*_max_ value of a series of S_N_2 reactions and predict the associated transition state structures. The S_N_2 reactions under consideration are as mentioned, with reactions of the following form: X^–^ + H_3_C–Y → X–CH_3_ + Y^–^, with X, Y ∈ {F, Cl, Br, I} and X ≠ Y. Again, the reward is computed using PaiNN. The MAEs for energies and forces are 0.87 and 0.20 kcal mol^–1^ Å^–1^, respectively, on a hold-out test set (see section S4.3 and Figure S3 of the Supporting Information for further details).

In contrast to the previous experiment, no initial sampling is done by the model prior to the training period; thus, the episodes commence initially from the geodesic interpolation, with again for computational efficiency, in each episode, 10 steps.

Looking at Figure 5a, in all cases, the reward is maximized and associated *F*_max_ is minimized. To give a comparative view on the effectiveness of the model, the transition structures produced by the model are compared to the reference structures. The structures are plotted on top of each other in Figure 5b. The structures obtained by quantum chemistry are transparent. As seen, the structures are in excellent agreement with each other. For quantitative measure, further computation of the root-mean-square deviations (RMSDs) is performed, which are shown below the images and are below 0.1 Å in all cases.

**Figure 5:**
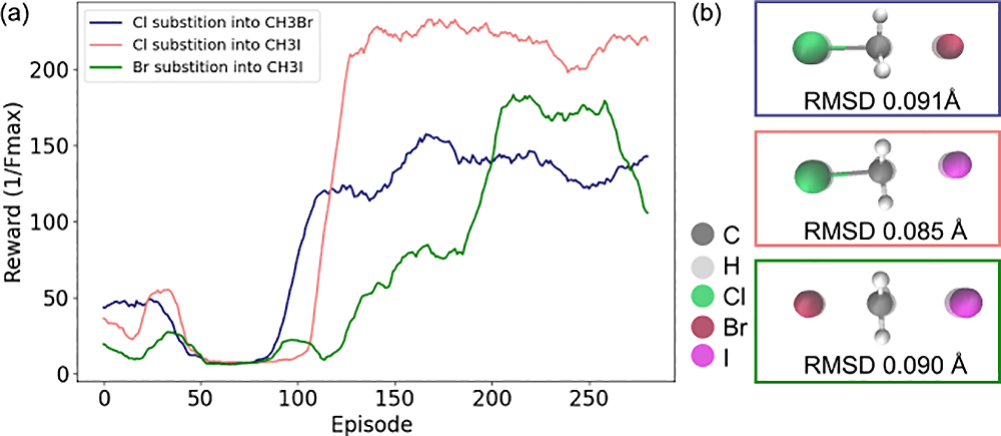
S_N_2 reactions. (a) Rewards through a training period of 300 episodes for a series of S_N_2 substitution reactions. (b) Transition states obtained with the model (solid) compared to transition states obtained with the original quantum chemical method (transparent), including RMSDs.

The test cases show the use of the model to predict energy pathways and transition states of a series of reaction mechanisms, as demonstrated for the organic allyl-*p*-tolyl ether Claisen rearrangement reaction and S_N_2 substitution reactions. The results show that the model produces transition states and corresponding energy curves, which closely resemble the quantum chemical reference values. In the case of the organic allyl-*p*-tolyl ether Claisen rearrangement reaction, in total, the actor–critic model took 60 steps to converge compared to the NEB with ML, which took 1341 steps. Furthermore, as a result of the exploratory nature of the reinforcement learning algorithm, it possesses the ability to search through large parts of the potential energy surface, whereas a standard NEB algorithm may be stuck in local minima, making the method particularly of interest to systems of high complexity, where standard NEB often fails. While the model can efficiently be trained on a single reaction, one drawback is that the training of the reinforcement learning algorithm is still more expensive than performing a standard NEB method with ML. However, this limitation becomes less pronounced with the more reactions that are trained on it.

Future research is needed to assess the performance on training of many diverse reactions at once. Further effort will thus be devoted to the development of expanding the model to generalize it more easily to a whole set of reactions. Additionally, further research will also look into integrating actor–critic methods into other molecular tasks. In addition, the restriction to hyperplanes generated by the internal and spring forces can be removed to allow for larger search space but with a larger computational cost. Because the reward function can be changed depending upon the use case to allow the actor–critic algorithm to target conformations with a certain set of properties, we expect that the reinforcement learning model has the potential to become a valuable tool for not only the estimation of transition states and minimum energy paths but also the advancement of the search for molecular conformations with target properties.

## Data Availability

The code is publicly available at https://github.com/rhyan10/_SchNebby_. The data set used for the Claisen rearrangement reaction is publicly available in ref (14) under the name ate_vacuum.tgz. The data set for the S_N_2 reactions is publicly available in ref (17).

## Supporting Information Available

Allyl-*p*-tolyl ether Claisen rearrangement reaction (XYZ)
Detailed description of the method and mathematical frameworks underlying the work, additional Figure S1 (critic architecture), Figure S2 (scatter plots of PaiNN versus reference energies and forces for the Claisen rearrangement reaction), Figure S3 (scatter plots of PaiNN versus reference energies and forces for the S_N_2 data set), and Figure S4 (actor architecture), and additional Table S1 (PaiNN hyperparameters), Table S2 (RL hyperparameters), and Table S3 (normal modes of the found transition state for the S_N_2 reactions) (PDF)
Transparent Peer Review report available (PDF)

## Supplementary Material

jz3c02771_si_001.xyz
