# Supplementary material for: Reinforcement Learning for Traversing Chemical Structure Space: Optimizing Transition States and Minimum Energy Paths of Molecules
Source: J Phys Chem Lett. 2024 Jan 3;15(1):349–56. doi: 10.1021/acs.jpclett.3c02771 (PMC10788951; doi:10.1021/acs.jpclett.3c02771)
Supplement: Supplementary file 2 — jz3c02771_si_002.pdf [file jz3c02771_si_002.pdf]

Supplementary Material for: Reinforcement Learning for Traversing Chemical Structure Space: Optimizing Transition States and Minimum Energy Paths of Molecules

Rhyan Barrett[a], Julia Westermayr[a,b,*]

[a] Institute of Chemistry, Faculty of Chemistry and Mineralogy, University of Leipzig, Johannisallee 29, 04103 Leipzig, Germany
[b] Center for Scalable Data Analytics and Artificial Intelligence (ScaDS.AI), Dresden/Leipzig, Humboldtstraße 25, 04105 Leipzig, Germany

[*] julia.westermayr@uni-leipzig.de

Contents

S1 Mathematical Description of Actor and Critic for Molecular Systems S2

S2 Loss Implementation S2

S3 Actor Implementation Details S4

S4 Critic Implementation Details S5

S5 Hyperparameters and Training S6

S5.1 PaiNN Model Training Details S6
S5.2 Claisen Rearrangement Datset Error Graph S7
S5.3 S_N2 Dataset Error Graph S8
S5.4 Model Architecture and Training Details S8

S6 Details of Implemented Nudged Elastic Band (NEB) MethodS10

S7 Quantum chemical reference calculations S12
S7.1 Frequency Calculations S13

S1 Mathematical Description of Actor and Critic for Molecular Systems

As discussed in the main paper, a short introduction into the mathematical details is given. Define R_i^t as the position of atom i in the molecule at step t in the episode then the position of atom i at time step t is sampled from the distribution $P(R_i^t|R_i^{t-1}, \pi(R_i^{t-1}|\theta))$, here $\pi(R_i^{t-1}|\theta)$ is the policy function approximated by the neural network parameterized by θ. The probability distribution for the next configuration in the episode at time step t can then be written as,

$$P(R^t|R^{t-1}) = \alpha \prod_{i=0}^{N_{atoms}} P(R_i^t|R_i^{t-1}, \pi(R_i^{t-1}|\theta)) \quad (1)$$

Here, α is the normalisation constant. The positions can then be sampled from the generated probability distributions to construct the new conformer. This process is repeated to form a Markov chain which constitutes the reinforcement learning algorithm.

To reiterate, the critic must estimate the long term rewards $V(S_k)$ but also be permutation invariant of the input by considering each atom individually then summing their associated contributions. Thus $V(S_k)$ can expanded as follows,

$$V(S_k) = \mathbb{E}(\mathbf{R_k}|S_k = R^k) = \mathbb{E}\left[\sum_{t=k}^{n} r_t|S_k = R^k\right] = \sum_{i=0}^{N_{atoms}} \mathbb{E}\left[\sum_{t=k}^{n} r_t^i|S_k = R^k\right]. \quad (2)$$

where summing over r_t^i corresponds to each atom's estimated contribution to the total reward at time step t. To avoid confusion $\mathbf{R_k}$ represents the cumulative rewards rather than positions. This structure allows easy implementation of the critic as a neural network for arbitrary-sized molecules.

S2 Loss Implementation

To reiterate from the paper $A_{k+n} > 0$ and $A_{k+n} < 0$ correspond to the associated action being positively and negatively reinforced, respectively. The

weight updates of a general actor are then constructed by weighting the policy gradients of each sample by their advantage value,

$$\nabla_\theta \mathcal{L}_a = \mathop{\mathbb{E}}_{\pi_\theta}[\nabla_\theta log \pi_\theta(a_k|S_k) A_{\pi_\theta}(a_k, S_k)] \tag{3}$$

Here a_k and A_k represent the action and advantage at time k respectively. The loss, $\mathcal{L}_c$ and weight updates for a general critic are calculated using a mean squared error (MSE) between the observed reward function and predicted value function $V(S_k)$:

$$\mathcal{L}_c = \left(\sum_{t=k}^{k+n} \gamma^t r_t + V(S_{k+n}) - V(S_k) \right)^2 \tag{4}$$

The training process in our actor-critic implementation is described as follows. The loss is computed using the advantage values obtained from the temporal difference targets (TD-targets). The total loss is equal to the sum of the actor and critic loss, $\mathcal{L}_a$ and $\mathcal{L}_c$, respectively:

$$\mathcal{L}_{total} = \mathcal{L}_a + \mathcal{L}_c \tag{5}$$

The total loss function for the actor, $\mathcal{L}_{policy}$ is calculated using an adapted version of the loss as mentioned below. The value $\mathcal{L}_{policy}$ weights the policy gradients calculated by the advantages of those actions. The entropy loss, *i.e.*, the second part in equation (4), encourages exploration by penalizing overly confident predictions. To this end we utilize a hyper-parameter β controlling the strength of the penalty of the entropy loss in order to control the amount of exploration.

$$\mathcal{L}_{policy}(action_{Pos}) = \sum_{k=1}^{samples} \left(\sum_{i=1}^{n} A_{k,i} \, log(Softmax(t_{k,i})) + \beta \sum_{i=1}^{n} t_{k,i} * log(Softmax(t_{k,i})) \right) \tag{6}$$

Here $t_{i,k}$ and $A_{k,i}$ corresponds to probability and advantage associated to the action at element k in the batch and at step i in the episode. Additionally, to generate an initial starting guess for the reaction pathway we use geodesic interpolation followed by high speed sampling using the model's initial distribution over the actions. This produces a guess much closer to the minimum energy pathway meaning less training time is wasted on pathways which are not useful. The critic is trained using standard temporal difference learning. In this case, the rewards are obtained from the $(F_{max})^{-1}$ values.

S3 Actor Implementation Details

Based on the representation mentioned in the paper, we aim to predict changes to the positions of atoms in each image along the reaction pathway at each step in order to move closer to the minimum energy pathway. To achieve changes in atomic positions, we look to generate distributions for positions changes around each atom. To elaborate further, around each atom in the molecule, we construct a fine discrete grid of α_i and β_i values to produce a linear combination of $F_{int} \perp$ and $F_{spr} \parallel$. A distribution is then constructed by the model on this grid and the new position is sampled from the respective distribution. It is important to note these α_i and β_i values can be generated per atom in each image or be the same for every atom in the image with the former being the most computationally intense. In the following section we will describe the case where an α_i and β_i value are generated for each individual atom.

In more detail, let us take the initial state S_t, of a reaction pathway, which contains the atomic types $\{Z_i\}$ and associated positions $\{\{R_{i_1}\}^1, \{R_{i_2}\}^2, ...,$ $\{R_{i_n}\}^n\}$ of all atoms in each molecule along the reaction pathway where n is the number of images and i_j corresponds to the index of the atom in image j. The probability distributions of the positions of the atoms in the reaction pathway at step T is given by $\{\{P(R_i)\}_j \; \forall j \in [0, n]\}$. To accommodate the auto-regressive nature of the reinforcement model we can write this as the product of conditional probabilities of the positions at each step which can be decomposed as changes in positions:

$$\{P(R_i^t)\}_j = \left[\prod_{t=0}^{T} \prod_{i=0}^{N_{atoms}} P(R_{i,j}^{t-1} + \delta R_{i,j}^t | R_{i,j-1}^{t-1}, R_{i,j}^{t-1}, R_{i,j+1}^{t-1})\right]_j \; \forall j \in [0, n]. \tag{7}$$

From now on we will abbreviate $R_{i,j-1}^{t-1}, R_{i,j}^{t-1}, R_{i,j+1}^{t-1}$ with $Y_{i,j}^{t-1}$. Additionally, we can decompose the distribution into the joint probability distribution of α_i and β_i values from which the changes in positions for each molecule along the reaction path are constructed:

$$\left[\prod_{t=0}^{T} \prod_{i=0}^{N_{atoms}} P(\delta R_{i,j}^t | Y_{i,j}^{t-1})\right]_j = \left[\prod_{t=0}^{T} \prod_{i=0}^{N_{atoms}} P(\alpha_i, \beta_i | Y_{i,j}^{t-1})\right]_j \; \forall j \in [0, n] \tag{8}$$

Furthermore, we add a dependency of β_i on the α_i value chosen. This leads

to the joint distribution being rewritten so that β_i depends on α_i;

$$\left[\prod_{t=0}^{T}\prod_{i=0}^{N_{atoms}} P(\alpha_i, \beta_i | Y_{i,j}^{t-1})\right]_j = \left[\prod_{t=0}^{T}\prod_{i=0}^{N_{atoms}} P(\beta_i | Y_{i,j}^{t-1}, \alpha_i) P(\alpha_i | Y_{i,j}^{t-1})\right]_j \quad \forall j \in [0, n]. \tag{9}$$

These distributions are produced as an output of the actor model and combined into a categorical distribution over a fine grid. A new position is then sampled from this grid for each atom in every image.

S4 Critic Implementation Details

As mentioned in the paper, the critic model employs identical PaiNN representation layers as the actor model, with the exception that the final layers are utilized to furnish an estimation of the value function

$$V^{\pi}(S_k) = \mathop{\mathbb{E}}_{\pi}(R_k | S_k = S) = \mathop{\mathbb{E}}_{\pi}\left[\sum_{t=k}^{\infty} \gamma^t r_t | S_k = S\right]. \tag{10}$$

At each step in the auto-regressive structure of the RL algorithm, the reward is given from the NEB calculation using the F_{max} quantity and a conventional PaiNN model. The atom and image of which F_{max} occurs in future steps is highly uncertain. However, it can be decomposed as the probability of each atom causing the largest force multiplied by the estimated force if this is the case; the expected F_{max} for a particular atom. More formally the value function decomposes as follows:

$$\mathop{\mathbb{E}}_{\pi}\left[\sum_{t=K}^{\infty} \gamma^t r_t | S_k = S\right] = \sum_{j=0}^{n}\sum_{i=0}^{N_{atoms}}\left[\sum_{t=k}^{\infty} \gamma^t \mathop{\mathbb{E}}_{\pi}(f_{max}^{z} | S_k = S)\right]. \tag{11}$$

Using the shared representation layer between the actor and critic models we then predict the expected values of each atom in each image, as it is shown in Figure S1. The resulting expectations are then summed in the final layers to give an estimate of the value function.

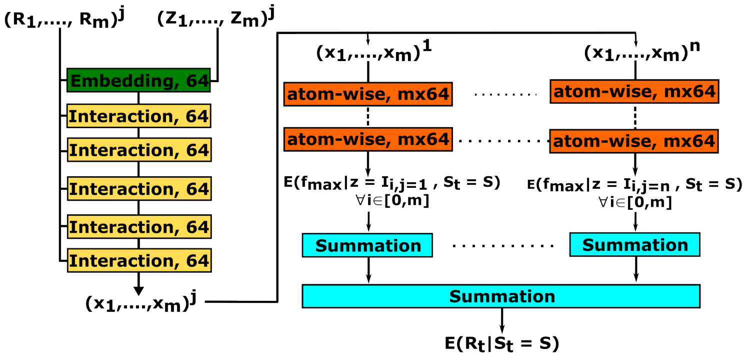

Figure S1: **Critic architecture.** The PaiNN model provides an initial feature representation, $(\mathbf{x_1}, ..., \mathbf{x_m})^j$ with j referring to the index of the image. For each image in the reaction path we pass the feature vectors for each atom through a series of atom-wise layers. The output contains the expectation of each atom in a molecule. The expectations are then summed over all atoms in each image and finally over all images to deliver the final expectation.

S5 Hyperparameters and Training

All experiments and trainings were run on an NVIDIA RTX A2000 12 GB GPU as well as 20 i7-12700 12th Gen CPUs.

S5.1 PaiNN Model Training Details

The datasets used for the training of the models are freely available and comprise the allyl-*p*-tolyl ether Claisen rearrangement reaction[5] as well as S_N2 reactions[15]. The data were generated with PBE0/def2-TZVP[1,17] and DSD-BLYP-D3(BJ)/def2-TZVP[6,9] level of theory, respectively. The target properties used for training of the Claisen rearrangement and S_N2 reactions were energy and forces. The loss weighting of energy and forces was 0.02 and 0.98, respectively, for both datasets. The dataset was split into training, validation, and test sets randomly. For the claisen rearrangement reaction, 50000 data points were used for training, 6000 for validation, and 5000 for testing. We used a batch size of 50. For the S_N2 dataset, 350000 data points were used for training, 50000 for validation, and 52709 for testing. A batch size of 500 was used.

For both datasets a cutoff function was applied. In addition, for the Claisen rearrangement reaction, the means of each target property were substracted from the dataset as well. Both models were built using SchNetpack[13] in combination with the equivariant PaiNN[12] representation. The hyperparameters for the model used in both the Claisen rearrangement and S_N2 reactions are as stated in Table 1. Different hyperparameters were

Table S1: Hyperparameters of the final PaiNN models trained on the Claisen rearrangement reaction and S_N2 reactions.

Hyperparameters of SchNetpack models		
Parameter	Claisen rearrangement	S_N2 Reaction
Representation	PaiNN	PaiNN
n_atom_basis	64	128
n_interactions	9	9
Radial basis function	Guassian	Guassian
n_rbf	20	20
cutoff_fn	CosineCutoff	CosineCutoff
cutoff	10	10
loss_fn	MSE	MSE
Optimizer	AdamW	AdamW
Learning rate	10^{-4}	10^{-4}

tested starting from the default values of PaiNN and changing them such that a good compromise between accuracy and computational efficiency could be achieved. In SchNetPack, interaction layers are followed by a series of atom-wise dense layers[14] where the following layer is compromised of half the number of neurons of the previous until 1 is reached, i.e. 64, 32, 16, 8, 4, 2, 1.

Figure S2 and S3 show the energies and forces in the test dataset against the energies and forces predicted by the PaiNN[12] models respectively.

S5.2 Claisen Rearrangement Datset Error Graph

The test set consists of 5000 data points. The original data set contains energies being measured in Hartree and forces measured in Hartree/Bohr. The mean absolute errors (MAEs) of energies and forces are 0.0003438 Ha and 0.000316 Ha/Bohr.

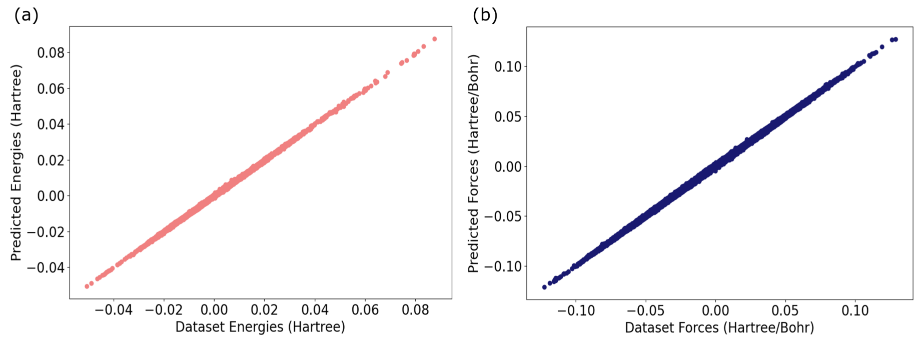

Figure S2: Claisen rearrangement reaction dataset: Scatter plot of a) energies and b) forces showing the test data against the values predicted by the PaiNN model.

S5.3 S_N2 Dataset Error Graph

The test set consists of 52709 datapoints with the energies being measured in eV and the forces measured in eV/Å. The MAEs of energies and forces are 0.03756 eV and 0.004517 eV/Å.

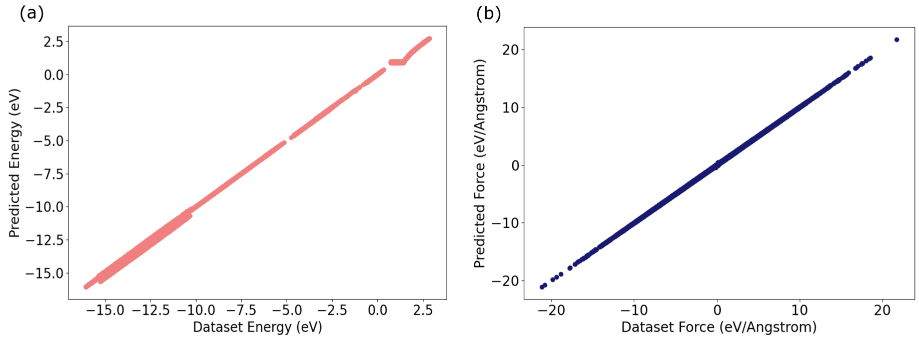

Figure S3: S_N2 dataset: Scatter plots of a) energies and b) forces showing the test dataset against the values predicted by the PaiNN model.

S5.4 Model Architecture and Training Details

The model utilizes the pretrained PaiNN representation for each dataset, which were introduced in the previous section, as an initial representation for the model. These representation vectors remain constant throughout the training episodes of the model.

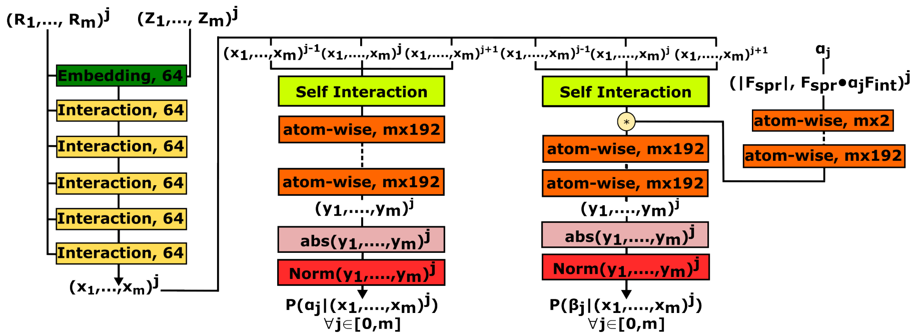

Figure S4: **Actor architecture.** The PaiNN representation forms the initial feature vectors for the actor. The positions of each atom in the current image are passed through a self interaction layer and a dense layer to produce the α_i distribution. A similar process is used to construct the associated β_i distribution. The two distributions are then sampled from to produce the change in positions at the current time step.

We show the actor architecture again in Figure S4 for better understanding of the following text. The self-interaction layers of the model are composed of a multi-attention head[16], with one head specifically designed to combine neighboring image representation vectors. The atom-wise dense layers in the model follow from the self-interaction layers, maintaining the same construction methodology discussed in the previous section; the following dense layer being composed of half the number of neurons in the previous layer. The output values were then sampled from the generated distributions. All energies and forces throughout were predicted using the PaiNN model from the previous section.

To streamline the performance, the spring force vector is used. The spring force vector is precalculated as an input into the model. In addition, the internal force vector is derived using the pretrained PaiNN model, serving as an additional input to the model. This incorporation of both force vectors reduces the computational time needed for training. For the Claisen rearrangement reaction and the S_N2 reactions, the model utilizes specific hyperparameters, which are provided in Table 2.

Table S2: Hyperparameters used for the model when training on the Claisen rearrangement reaction and S_N2 reactions.

Hyperparameters of the model		
Parameter	Claisen rearrangement reaction	S_N2 reactions
Batch size	10	20
Grid size	10×10	10×10
α, β range	100	3
Spring constant (k)	0.1	0.1
Learning rate	10^{-4}	10^{-4}
Preoptimise length	50	0
Episode length	10	10
Optimizer	Adam	Adam

During the training period two pathways found by the model are stored. These are the pathway corresponding to the minimum activation energy and minimum F_{max} found through all episodes. Due to the stochastic sampling procedure it is worth to mention that results are likely not reproducible exactly, but within a certain statistical accuracy. For better reproducibility, however, the associated trained PaiNN models are provided in addition to this document and code.

S6 Details of Implemented Nudged Elastic Band (NEB) Method

The reaction pathway is a crucial concept in understanding chemical reactions. It provides insights into the sequence of structural changes occurring between the initial and final states of a reaction. In order to construct a reaction pathway, a method called the nudged elastic band (NEB)[8] is commonly used.

The reaction pathway is formed initially from the start and end structures and using a series of $N + 1$ images connected by virtual spring forces forming a sequence of connected structures. The sequence with $N + 1$ images can be written as $\{\mathbf{R}_0, \mathbf{R}_1, ..., \mathbf{R}_{n-1}, \mathbf{R}_n\}$ where $\mathbf{R}_0$ and $\mathbf{R}_n$ are the initial and final structures respectively. The initial and final images correspond to specific molecular configurations, which correspond to local energy minima. The

positions of the endpoints are thus held fixed to maintain a constant energy throughout the process.

To connect these images and simulate the structural changes along the pathway, virtual spring forces are introduced. The key component of these spring forces is the tangent vector, denoted as τ_i, which describes the direction of the pathway at each image. Initially, a simple estimate of the tangent vector can be obtained by calculating the normalized line segments between neighboring images and summing them:

$$\tau_i = \frac{\mathbf{R}_i - \mathbf{R}_{i-1}}{|\mathbf{R}_i - \mathbf{R}_{i-1}|} + \frac{\mathbf{R}_{i+1} - \mathbf{R}_i}{|\mathbf{R}_{i+1} - \mathbf{R}_i|} \tag{12}$$

However, this basic approach can lead to the formation of kinks or irregularities in the pathway. More information about this can be found in reference 8. To address this issue, a more sophisticated method is proposed. Let i_{max} be the index of the image with the maximum potential energy; this corresponds to the transition state.

$$\begin{aligned}
\tau_i^- &= \frac{\mathbf{R}_i - \mathbf{R}_{i-1}}{|\mathbf{R}_i - \mathbf{R}_{i-1}|} \quad i < i_{max} \\
\tau_i^+ &= \frac{\mathbf{R}_{i+1} - \mathbf{R}_i}{|\mathbf{R}_{i+1} - \mathbf{R}_i|} \quad i > i_{max} \\
\tau_{i_{max}} &= \frac{\mathbf{R}_i - \mathbf{R}_{i-1}}{|\mathbf{R}_i - \mathbf{R}_{i-1}|} + \frac{\mathbf{R}_{i+1} - \mathbf{R}_i}{|\mathbf{R}_{i+1} - \mathbf{R}_i|} \quad i = i_{max}
\end{aligned} \tag{13}$$

With the tangent vectors established, the respective spring forces between neighboring images can be calculated. Additionally these forces are parameterized by a spring constant, denoted as k, which quantifies the strength of the interaction between neighboring images. The spring force denoted as $\mathbf{F}^i_{spr} \parallel$, can be expressed as:

$$\mathbf{F}^i_{spr} \parallel = k(|\mathbf{R}_{i+1} - \mathbf{R}_i| - |\mathbf{R}_i - \mathbf{R}_{i-1}|)\tau_i \tag{14}$$

Additionally, the perpendicular force, denoted as $\mathbf{F}_{int} \perp$, which arises from internal molecular forces is considered. This term is defined as the internal force vector that acts orthogonal to the spring force vector. The combined force acting on each image, denoted as $\mathbf{F}^i$, is then defined as the sum of the spring force and the internal molecular force acting perpendicular to the pathway:

$$\begin{gathered}
\mathbf{F}^i = \mathbf{F}^i_{spr} \parallel + \mathbf{F}_{int} \perp \\
\text{with } \mathbf{F}_{int} \perp = \nabla E(R_i) \perp = \nabla E(R_i) - \nabla E(R_i) \parallel .
\end{gathered} \tag{15}$$

After each episode, molecular positions are then optimised using an optimisation algorithm. In the NEB experiments performed optimisation was done using BFGS[18]. By applying the BFGS algorithm to the force vector, one can iteratively adjust the positions of the images along the reaction coordinate, seeking the minimum energy pathway. The algorithm uses the current force vector and the approximated Hessian matrix to determine the search direction and step length for each iteration. The positions of the images are updated accordingly, aiming to converge towards a pathway with lower reaction barrier. More information on this can be found in 18. The model constructs a new forces vector with the predicted α_i and β_i values;

$$F^i = \alpha_i F_{int} \perp + \beta_i F^i_{spr} \parallel \tag{16}$$

one step is then taken with the BFGS algorithm to generate the new positions given the relevant force vectors. The BFGS algorithm and script to calculate F^i values given the α_i and β_i used the Atomic Simulation Environment (ASE).[10]

S7 Quantum chemical reference calculations

All quantum chemical calculations were conducted using the ORCA 5.0.3[11] program suite. For SCF cycles the convergence levels were set to tight SCF and the integration grid was set to defgrid2. In the dataset for the Claisen rearrangement reaction all calculations were conducted using the TZVP basis set and the PBE0[1] functional. For the S_N2 dataset all calculations were performed with the TZVP basis set, the DSD-BLYP functional[9] and the D3BJ[6] dispersion correction. The details for methods used in constructing the respective datasets for the Claisen rearrangement reaction and S_N2 reactions can be found at[5] and[15] respectively.

The minimum energy pathway for the Claisen rearrangement reaction was calculated using the NEB implementation in ORCA. Initially the NEB was run with the PBE functional[4] and SVP basis set. This pathway was used as a starting point for the following NEB. This NEB was done using the TZVP basis set with the PBE0 functional and D4 dispersion correction[3]. The NEB calculations were run using the climbing image implementation[7].

To calculate the root mean squared deviation (RMSD) between the generated transition states and the reference transition states we did a transition state optimisation with DFT. For the S_N2 reactions, predicted transition states from the model were taken as the initial starting points for transition state optimisation. An eigenvalue following algorithm was applied

using OptTS option in ORCA. The TZVP basis set was used along with the RI-C auxiliary basis set[2]. Again the DSD-BLYP functional and the D3BJ dispersion correction were used.

S7.1 Frequency Calculations

The frequency calculations of generated structures were performed using the DSD-BLYP functional, D3BJ dispersion correction, TZVP basis set and RI-C auxiliary basis set. The frequency calculations were computed numerically. The associated frequencies for each of the S_N2 reactions of the following form can be seen in the table below.

$$X^{-} + H_3C\text{–}Y \rightarrow X\text{–}CH_3 + Y^{-} \quad X, Y \in \{F, Cl, Br, I\}$$

Table S3: Normal modes of the found transition state for the S_N2 reactions.

S_N2 Reactions Frequencies			
Degrees of Freedom	$X = Cl$, $Y = Br$ [cm^{-1}]	$X = Cl$, $Y = I$ [cm^{-1}]	$X = Br$, $Y = I$ [cm^{-1}]
0	0.0	0.0	0.0
1	0.0	0.0	0.0
2	0.0	0.0	0.0
3	0.0	0.0	0.0
4	0.0	0.0	0.0
5	0.0	0.0	0.0
6	-795.92	-718.09	-604.47
7	142.54	132.18	104.66
8	149.33	157.69	134.27
9	150.75	160.16	137.94
10	791.66	801.02	801.50
11	792.68	802.21	803.23
12	895.63	907.19	885.53
13	1387.45	1392.63	1390.87
14	1387.92	1394.92	1391.26
15	3352.51	3336.25	3338.38
16	3575.85	3553.72	3560.16
17	3577.45	3556.60	3560.77

The frequency calculation for the transition state of the Claisen rearrangement reaction generated by the model was performed with the TZVP basis

set and the PBE0 functional. As with the S_N2 reaction all frequencies were computed numerically. The molecules has 63 normal modes hence results will be provided in an additional document. The structure has 6 negative frequencies, which reveals that the structure is not exactly the transition state. However, most frequencies are very small in their magnitude, i.e., only two are smaller than -300, which indicates that the structure is close to the transition state. In fact, many transition states found with NEB and quantum chemistry reference methods often comprise of more than one negative frequency. The RMSD of the calculated transition state and the transition state obtained with NEB using DFT is about 0.79 Åwhich is reasonable given the many degrees of freedom of the molecule.

References

[1] Carlo Adamo and Vincenzo Barone. Toward reliable density functional methods without adjustable parameters: The pbe0 model. *J. Chem. Phys.*, 110(13):6158–6170, 1999.

[2] Francesco Aquilante, Roland Lindh, and Thomas Bondo Pedersen. Unbiased auxiliary basis sets for accurate two-electron integral approximations. *J. Chem. Phys.*, 127(11):114107, 2007.

[3] Eike Caldeweyher, Sebastian Ehlert, Andreas Hansen, Hagen Neugebauer, Sebastian Spicher, Christoph Bannwarth, and Stefan Grimme. A generally applicable atomic-charge dependent london dispersion correction. *J. Chem. Phys.*, 150(15):154122, 2019.

[4] Matthias Ernzerhof and Gustavo E Scuseria. Assessment of the perdew–burke–ernzerhof exchange-correlation functional. *J. Chem. Phys.*, 110(11):5029–5036, 1999.

[5] Michael Gastegger, Kristof T Schütt, and Klaus-Robert Müller. Machine learning of solvent effects on molecular spectra and reactions. *Chem. Sci.*, 12(34):11473–11483, 2021.

[6] Lars Goerigk. A comprehensive overview of the dft-d3 london-dispersion correction. *Non-Covalent Interactions in Quantum Chemistry and Physics*, pages 195–219, 2017.

[7] Graeme Henkelman, Blas P Uberuaga, and Hannes Jónsson. A climbing image nudged elastic band method for finding saddle points and minimum energy paths. *J. Chem. Phys.*, 113(22):9901–9904, 2000.

[8] Hannes Jónsson, Greg Mills, and Karsten W Jacobsen. Nudged elastic band method for finding minimum energy paths of transitions. In *Classical and quantum dynamics in condensed phase simulations*, pages 385–404. World Scientific, 1998.

[9] Sebastian Kozuch, David Gruzman, and Jan ML Martin. Dsd-blyp: A general purpose double hybrid density functional including spin component scaling and dispersion correction. *J. Phys. Chem. C.*, 114(48):20801–20808, 2010.

[10] Ask Hjorth Larsen, Jens Jørgen Mortensen, Jakob Blomqvist, Ivano E Castelli, Rune Christensen, Marcin Dułak, Jesper Friis, Michael N Groves, Bjørk Hammer, Cory Hargus, et al. The atomic simulation environment—a python library for working with atoms. *J. Phys. Condens.*, 29(27):273002, 2017.

[11] Frank Neese. The orca program system. *WIREs: Comput. Mol. Sci.*, 2(1):73–78, 2012.

[12] Kristof Schütt, Oliver Unke, and Michael Gastegger. Equivariant message passing for the prediction of tensorial properties and molecular spectra. In *International Conference on Machine Learning*, pages 9377–9388. PMLR, 2021.

[13] Kristof T Schütt, Stefaan SP Hessmann, Niklas WA Gebauer, Jonas Lederer, and Michael Gastegger. Schnetpack 2.0: A neural network toolbox for atomistic machine learning. *arXiv:2212.05517*, 2022.

[14] Kristof T Schütt, Huziel E Sauceda, P-J Kindermans, Alexandre Tkatchenko, and K-R Müller. Schnet–a deep learning architecture for molecules and materials. *Chem. Phys.*, 148((24)), 2018.

[15] Oliver T Unke and Markus Meuwly. Physnet: A neural network for predicting energies, forces, dipole moments, and partial charges. *J. Chem. Theory Comput.*, 15(6):3678–3693, 2019.

[16] Ashish Vaswani, Noam Shazeer, Niki Parmar, Jakob Uszkoreit, Llion Jones, Aidan N Gomez, Łukasz Kaiser, and Illia Polosukhin. Attention is all you need. *Advances in neural information processing systems*, 30, 2017.

[17] Florian Weigend and Reinhart Ahlrichs. Balanced basis sets of split valence, triple zeta valence and quadruple zeta valence quality for h

to rn: Design and assessment of accuracy. *Phys. Chem. Chem. Phys.*, 7(18):3297–3305, 2005.

[18] Ya-xiang Yuan. A modified bfgs algorithm for unconstrained optimization. *IMA J. Numer. Anal.*, 11(3):325–332, 1991.
