# Supplementary material for: Reinforcement Learning for Traversing Chemical Structure Space: Optimizing Transition States and Minimum Energy Paths of Molecules
Source: J Phys Chem Lett. 2024 Jan 3;15(1):349–56. doi: 10.1021/acs.jpclett.3c02771 (PMC10788951; doi:10.1021/acs.jpclett.3c02771)
Supplement: Supplementary file 3 — jz3c02771_si_003.pdf [file jz3c02771_si_003.pdf]

jz-2023-027719.R1

Name: Peer Review Information for "Reinforcement Learning for Traversing Chemical Structure Space: Optimizing Transition States and Minimum Energy Paths of Molecules"

First Round of Reviewer Comments

Reviewer: 1

Comments to the Author

In their publication, Barrett et al. present a machine-learning algorithm based on actor-critic reinforcement learning that facilitates the optimisation of molecular structures to attain desired properties. The authors demonstrate the validity of their approach by modelling a number of reaction pathways. Given that reinforcement learning has recently seen interest in the quantum chemistry community, the current research is a valuable addition to the field. The manuscript is well written, the science is adequately explained and all figures are legible. However, a few improvements should be made to the current manuscript.

Minor comments:

1. The work would benefit from a discussion of the existing literature on reinforcement learning in the field of quantum chemistry. There exist a number of implementations of deep reinforcement learning, multi-agent reinforcement learning, or Q-learning that are dedicated to molecular/crystal/surface structure determination.

2. Atom positions are referred to only as positions, which is a bit confusing in the beginning.

3. The work would benefit from a (more detailed) comparison to other implementations of ML-NEB. The number of required training data should be compared to other methods. (I could only find the numbers for the new approach in the SI)

4. Figure 3 should be presented in a clearer way and the different variables should be explained in the figure caption. Moreover, there is a typo in the caption of Figure 3.

Reviewer: 2

Comments to the Author

The manuscript by Barrett and Westermayr describes how actor-critic reinforcement learning (ac-RL) can be utilized to calculate minimum energy paths of chemical reactions.

While the ac-RL technique certainly deserves its position in the theoretical chemist's toolbox, I consider the work too technical and long for a letter, and at the same time too short to understand the authors' approach in detail. In my assessment, even the average reader with machine learning experience will find it difficult to follow the explanations. What would be needed is a comprehensive foundational technical work by the authors that they could then refer to in a letter. Also, the presentation does not address the readership of the journal. No chemistry-specific research is discussed until page 10 (!).

What I am also missing is a critical assessment of the developed method with respect to rather conventional quantum chemical calculations of minimum energy paths. What is the efficiency gain of the method? Are there further advantages? For instance, do the authors expect ac-RL to replace NEB calculations in the long run?

Minor issues:

- p. 8: Fig. 3b is cited, but it does not exist.

- Symbols are only explained later in the text, which makes it difficult to initially understand the text. In some cases, I cannot even find definitions for symbols, such as s and θ in the expression π(s|θ).

- The model abbreviation PaiNN is introduced without definition or explanation. I do not consider this term a community standard.

In summary: I am confident that the work is of high potential value to the community, but it is hardly digestible in its current form.

Author's Response to Peer Review Comments:

We thank the reviewers for their critical evaluation of our manuscript and insightful comments to our work. These are addressed in the attached file.

Reply to reviewer comments

We thank the reviewers for their critical evaluation of our manuscript and insightful comments to our work. Below we address each of the comments and suggestions provided by the reviewers and highlight the modifications we incorporate in the revised manuscript. The revised texts are presented in blue and red color for better visibility.

Reviewer: 1

Comments to the Authors:
In their publication, Barrett et al. present a machine-learning algorithm based on actor-critic reinforcement learning that facilitates the optimisation of molecular structures to attain desired properties. The authors demonstrate the validity of their approach by modelling a number of reaction pathways. Given that reinforcement learning has recently seen interest in the quantum chemistry community, the current research is a valuable addition to the field. The manuscript is well written, the science is adequately explained and all figures are legible. However, a few improvements should be made to the current manuscript.

Minor comments:

Response: We thank the reviewer for their positive evaluation of our work.

Comment 1: The work would benefit from a discussion of the existing literature on reinforcement learning in the field of quantum chemistry. There exist a number of implementations of deep reinforcement learning, multi-agent reinforcement learning, or Q-learning that are dedicated to molecular/crystal/surface structure determination.

Response 1: We revised and added additional material to introduce some previous works in the area of molecular design, crystal and surface determination.

Comment 2: Atom positions are referred to only as positions, which is a bit confusing in the beginning.

Response 2: We have adjusted this in the manuscript to make this clearer.

Comment 3: The work would benefit from a (more detailed) comparison to other implementations of ML-NEB. The number of required training data should be compared to other methods. (I could only find the numbers for the new approach in the SI)

Response 3: We have added an additional analysis at the end of the paper explaining the current pros and limitations of the proposed method. In the case of the training data a pretrained PaiNN model is used in either case so the training data is the same, independent of NEB (with ML) or the reinforcement learning implementation is used. The advantages being that on large datasets we would hope that our method would converge much faster as shown by the number of steps of the trained reinforcement model vs the standard NEB method. Additionally reinforcement learning has the ability to explore large parts of the potential energy surface making it useful for reactions where standard NEB does not converge. The drawback being that the reinforcement learning model requires additional training time making it less beneficial for single reactions. We added the following discussion to the text:

"…. In the case of the organic allyl-p-tolyl ether Claisen rearrangement reaction, in total the actor-critic model took 60 steps to converge compared to the NEB with ML which took 1341 steps. Furthermore, due to the exploratory nature of the reinforcement learning algorithm it possess the ability to search through large parts of the potential energy surface where as a standard NEB algorithm may be stuck in local minima making the method particularly of interest to systems of high complexity, where standard NEB often fails. While the model can efficiently be trained on single reaction one drawback is that the training of the reinforcement learning algorithm is still more expensive than performing a standard NEB method with ML. However, this limitation becomes less pronounced the more reactions are trained on. …"

Comment 4: Figure 3 should be presented in a clearer way and the different variables should be explained in the figure caption. Moreover, there is a typo in the caption of Figure 3.

Response 4: We have adapted the image in order to reduce its technically and moved the highly detailed image to the supplementary material. We attach both images below.
New image:

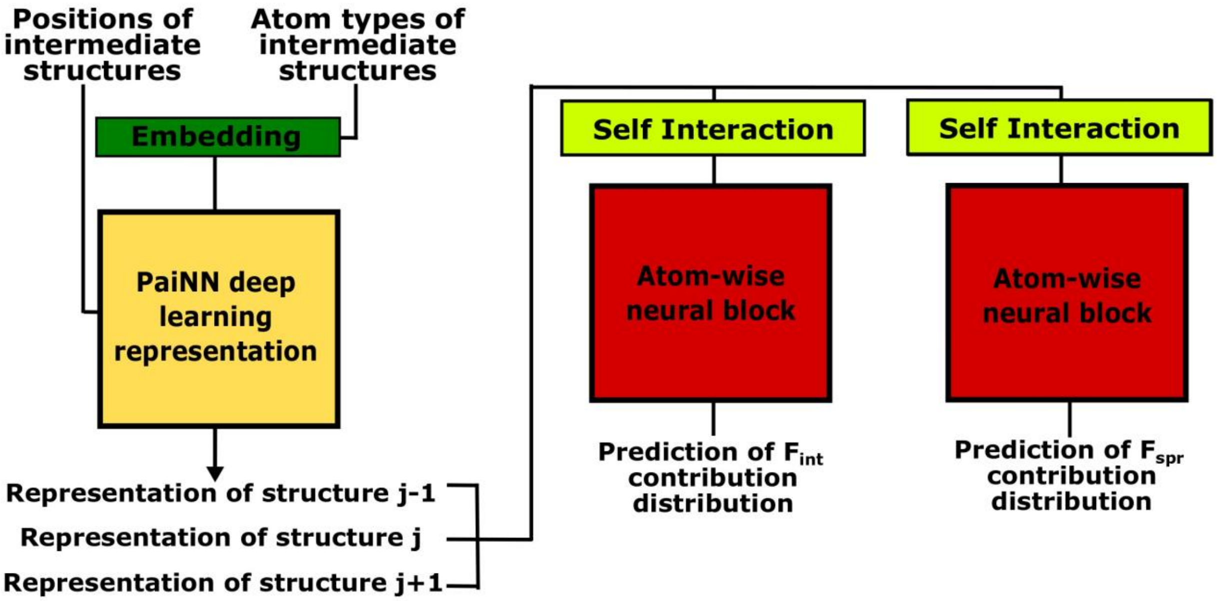

Previous image (now Fig. S4):

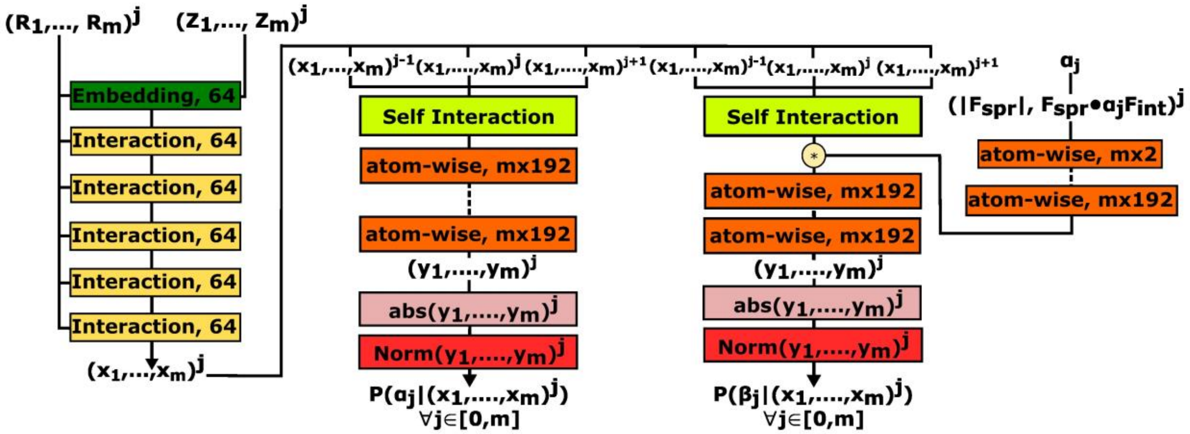

Reviewer: 2

Comments to the Authors:
The manuscript by Barrett and Westermayr describes how actor-critic reinforcement learning (ac-RL) can be utilized to calculate minimum energy paths of chemical reactions.

While the ac-RL technique certainly deserves its position in the theoretical chemist's toolbox, I consider the work too technical and long for a letter, and at the same time too short to understand the authors' approach in detail. In my assessment, even the average reader with machine learning experience will find it difficult to follow the explanations. What would be needed is a comprehensive foundational technical work by the authors that they could then refer to in a letter. Also, the presentation does not address the readership of the journal. No chemistry-specific research is discussed until page 10 (!).

What I am also missing is a critical assessment of the developed method with respect to rather conventional quantum chemical calculations of minimum energy paths. What is the efficiency gain of the method? Are there further advantages? For instance, do the authors expect ac-RL to replace NEB calculations in the long run?

Response: We appreciate the reviewer's feedback on our manuscript describing the application of actor-critic reinforcement learning. We understand the concern that the manuscript may be overly technical and lengthy for a letter format. However, we would like to clarify that the depth and technicality of the manuscript stem from the nature of the topic. The utilization of ac-RL in the domain of theoretical chemistry is a novel and intricate field, and it requires a foundation for readers to grasp its implications fully. We agree with the reviewer's suggestion that a foundational technical work could be developed, which could serve as a reference for future letters and more accessible articles. We will look into writing something like this specific to molecular tasks but we want to note that literature already exists as background for reinforcement learning. In addition, we would like to mention that the technical details are all described in the supplementary material, which we believe will provide a foundational understanding of the subject area and could be seen as a "comprehensive foundational technical work".

Moreover, we acknowledge the reviewer's point about the limited discussion of chemistry-specific research until a later section of the manuscript. We have restructured the paper to present relevant chemistry-specific aspects earlier in the text by removing some of the mathematical technicality. Some of it is moved to the supporting information, some of it is simplified, as can be seen by the colored text.
However, due to the nature of the paper we still feel it is important to introduce the methodology in the early parts of the paper since these are the main ideas. Additionally, we appreciate the suggestion to provide

a critical assessment of the ac-RL method compared to conventional quantum chemical calculations of minimum energy paths. In our revised manuscript, we incorporated a more explicit analysis of the method's efficiency gains and its potential advantages in comparison to traditional techniques in the conclusion (see also comment 3 by reviewer 1). In the context of ac-RL (actor-critic reinforcement learning), it might be considered a potential future strategy if the community collectively trained an agent on a wide range of chemical reactions, ultimately replacing the traditional NEB method. One significant challenge with reinforcement learning is its high data requirements, but FAIR principles and the amount of data generated are definitely beneficial. Nevertheless, the latter fact makes it challenging to use the method as a readily available method when dealing with limited data. However, if a model is trained on a substantial dataset of reactions, it has the potential to outperform the standard NEB method. Moreover, in cases where NEB struggles to converge, reinforcement learning could offer a solution due to its capacity to explore a large portion of the potential energy surface during training.
In addition, we simplified the network architecture (actor-image) and moved all details to the SI.

Minor Comments:

Comment 1: p. 8: Fig. 3b is cited, but it does not exist.

Response 1: We have corrected this and cited the correct Figure.

Comment 2: Symbols are only explained later in the text, which makes it difficult to initially understand the text. In some cases, I cannot even find definitions for symbols, such as s and θ in the expression $\pi(s|\theta)$.

Response 2: We have again adjusted the paper so that it contains only the core aspects of the actor-critic method and thus it is more readable for physical chemists.

Comment 3: The model abbreviation PaiNN is introduced without definition or explanation. I do not consider this term a community standard.

Response 3: PaiNN is a machine learning potential for predicting energies and forces which is used in conjunction with the reinforcement learning algorithm. We have adjusted the text in the paper to make this clear. We added the following sentence: “PaiNN is a polarizable atom interaction neural network that learns equivariant representations in addition to the relation of these features to output targets.”

jz-2023-027719.R2

Name: Peer Review Information for "Reinforcement Learning for Traversing Chemical Structure Space: Optimizing Transition States and Minimum Energy Paths of Molecules"

Second Round of Reviewer Comments

Reviewer: 2

Comments to the Author

The authors have thoroughly taken the comments by both reviewers into account. The significant improvement of the revised manuscript are:

- clearer presentation of the technical details
- a more chemistry-specific discussion of the research
- a critical performance assessment of the proposed method with respect to established quantum chemical/ML approaches

Through these improvements, I am confident that the research will be of interest to a substantial number of physical chemists—and not only because of its problem-agnostic character.

While the research is now publishable in my opinion, I still feel that the work is too comprehensive for a letter.

Author's Response to Peer Review Comments:

Dear Editor,

thanks lot for considering our article. We are very happy about the decision and addressed the non-scientific comments as requested.

Best regards,

Julia
